# Two siblings presented with spastic paraplegia and psychomotor retardation with or without seizures with HACE1 variants: A case report and literature review

**DOI:** 10.1097/MD.0000000000051037

**Published:** 2026-10-09

**Authors:** Jiong Deng, Yuheng Gao

**Affiliations:** a Children’s Medical Center, Peking University First Hospital, Beijing, China.

**Keywords:** case report, compound heterozygous variants, HACE1, neurodevelopmental disorder, spastic paraplegia and psychomotor retardation with or without seizures

## Abstract

**Rationale:**

Spastic paraplegia and psychomotor retardation with or without seizures (SPPRS) is a rare autosomal recessive neurodevelopmental disorder caused by biallelic *HACE1* variants. Its long-term clinical course and genotype–phenotype spectrum remain incompletely characterized.

**Patient concerns:**

Two siblings from a nonconsanguineous Chinese family presented with infantile-onset hypotonia, global developmental delay, markedly delayed motor milestones, gait impairment, language delay, and dysarthria. The older sister experienced recurrent febrile seizures, whereas her younger brother had no seizures.

**Diagnoses:**

Whole-exome sequencing followed by Sanger validation identified compound heterozygous HACE1 variants in both siblings: c.1303C > T (p.Gln435Ter), inherited from the father, and c.2158_2159insG (p.Glu720GlyfsTer17), inherited from the mother. Both variants were considered disease-causing after clinical, segregation, database, and ACMG-based assessment.

**Interventions:**

Both patients received symptomatic rehabilitation and underwent long-term neurological and developmental follow-up.

**Outcomes:**

At the last follow-up, at 25 and 16 years of age, respectively, both siblings could walk independently with a waddling and unstable gait but remained unable to run. Both could understand basic instructions and communicate using short phrases, although dysarthria and substantial cognitive impairment persisted. An updated literature review demonstrated marked clinical heterogeneity among patients with *HACE1*-associated SPPRS, with hypotonia, developmental impairment, spasticity, speech disturbance, seizures, and structural brain abnormalities occurring at variable frequencies.

**Lessons:**

These cases expand the genotypic spectrum of *HACE1*-associated SPPRS and demonstrate that a relatively mild and gradually improving clinical course may occur. Early molecular diagnosis can facilitate individualized rehabilitation, genetic counseling, and prenatal diagnostic planning.

## 1. Introduction

Spastic Paraplegia and Psychomotor Retardation with or without Seizures (SPPRS; OMIM 616756) is a rare autosomal recessive neurodevelopmental disorder. First reported by Hollstein et al and Akawi et al in 2015,^[1,2]^ only a limited number of cases have been documented globally. The causative gene is *HACE1* (HECT domain and ankyrin repeat containing E3 ubiquitin protein ligase 1), which encodes an E3 ubiquitin ligase of the HECT family widely expressed in various tissues. SPPRS typically presents in infancy with hypotonia, progressing to severe motor impairment and global neurodevelopmental delay. Clinical features often include progressive bilateral lower limb spasticity and language impairment, with phenotypic variability. Magnetic Resonance Imaging (MRI) typically reveals cerebral atrophy, hypoplasia, and reduced white matter volume. This study reports the clinical phenotypes, imaging characteristics, and genetic analysis of 2 siblings with *HACE1* mutations in a Chinese family, accompanied by a review of the relevant literature.

## 2. Materials and methods

### 2.1. Study design and patients

This study was designed as a pedigree-based case report with a literature review. Two siblings from a nonconsanguineous Chinese family were evaluated at the Children’s Medical Center Outpatient Clinic of Peking University First Hospital because of unexplained developmental delay. Clinical data were collected from medical records and supplemented through long-term outpatient and telephone follow-up. The collected information included perinatal history, developmental milestones, neurological manifestations, seizure history, motor and language development, visual and hearing status, rehabilitation history, neuroimaging findings, laboratory investigations, genetic testing results, and functional status at the last follow-up.

The study was approved by the Medical Ethics Committee of Peking University First Hospital (#2005 [004]). Written informed consent was obtained from the parents of the children.

### 2.2. Clinical, neuroimaging, and laboratory assessment

Both patients underwent detailed neurological evaluation. Brain MRI was performed as part of the etiological assessment. Laboratory investigations included metabolic screening of blood amino acids, acylcarnitines, and urine organic acids, as well as routine blood tests, biochemical tests, liver and kidney function tests, immune profiles, erythrocyte sedimentation rate, and myocardial enzyme testing. Methylation-specific multiplex ligation-dependent probe amplification for Prader–Willi syndrome and Angelman syndrome, karyotype analysis, and copy number variant analysis were also performed to exclude other possible causes of developmental delay.

### 2.3. Genetic analysis

WES was performed in 2019 for the 2 siblings and their parents by Beijing Chigene Translational Medical Research Center. Genomic DNA was captured using the IDT xGen Exome Research Panel v1.0 and sequenced on an Illumina NovaSeq 6000 platform, with sequencing coverage of ≥ 99%. Variants were filtered and annotated using a cloud-based precision diagnosis system integrating molecular annotation and clinical phenotype analysis. Public and clinical databases, including OMIM, HGMD, ClinVar, gnomAD, and dbSNP, were used for variant interpretation. Candidate variants were classified according to the American College of Medical Genetics and Genomics guidelines and were validated by Sanger sequencing. Sequence analysis and protein domain visualization were performed using DNASTAR software.

### 2.4. Literature search and data extraction

A literature search was conducted in PubMed, Wan fang Data, CNKI, and VIP databases for articles published from 2015 to 2025. The search terms included “SPPRS,” “spastic paraplegia and psychomotor retardation with or without seizures,” and “HACE1.” Articles were eligible if they reported patients with genetically confirmed *HACE1*-associated SPPRS and provided individual-level clinical or genetic information. Duplicate publications, review articles without original cases, and studies without sufficient clinical or molecular data were excluded.

The following data were extracted when available: sex, nationality, family history, consanguinity, age at onset, initial symptoms, motor development, language and cognitive development, seizure history, ocular and auditory findings, cranial imaging findings, skeletal or dysmorphic features, HACE1 variant type, affected protein domain, zygosity, and clinical outcome.

### 2.5. Data synthesis

Because of the rarity of *HACE1*-associated SPPRS and the heterogeneity of the reported cases, no meta-analysis was performed. Clinical and genetic features were summarized descriptively. Categorical variables were presented as numbers and percentages, and follow-up age was summarized using median and range when available.

## 3. Results

### 3.1. Clinical manifestations

Proband: The proband is a female, aged 9 years at the time of initial presentation. She first presented in December 2010 with a chief complaint of “developmental delay since childhood.” She exhibited generalized hypotonia and global developmental delay from infancy.

Developmental milestones were significantly delayed: head control at 1 year, independent sitting at 2 years, independent standing at 4 years, and independent walking at 4.5 years. Her gait was waddling and unstable; at age 7, she could squat and stand but had poor balance and could not run. Language development was also delayed: she produced reduplicated words (e.g., “mama”) at 1.5 years and simple 5~6 word sentences by age 6, though with dysarthria. She could understand simple sentences and memorize short poems but lacked arithmetic skills. Her history included 8 to 10 episodes of febrile seizures, which ceased after age 8. There was no history of dysphagia or visual/hearing impairment.

Personal and Family History: She was the first child (G1P1), born full-term via cesarean section due to dystocia with a birth weight of 3.3 kg. There was a history of birth asphyxia, but no feeding difficulties. The pregnancy was uneventful. The parents are non-consanguineous.

Diagnosis and Follow-up: The initial clinical diagnosis was “developmental delay” of undetermined etiology. She received symptomatic treatment, including rehabilitation training. Long-term follow-up was conducted via outpatient visits and telephone interviews. At the last follow-up (age 25 years), she showed gradual improvement. She could walk independently with a waddling, unstable gait but remained unable to run. She understood basic sentences and could engage in simple communication.

Proband’s Brother: The younger brother presented in December 2010 at 5 months of age with “developmental delay since childhood.” His clinical presentation mirrored the proband’s, characterized by generalized hypotonia and global developmental delay. At presentation, he had poor head control, could not crawl or roll over, and did not actively grasp objects. Visual tracking and social smiling were poor. He had no history of seizures, dysphagia, or sensory impairment.

Personal History: He was the second child (G2P2), born full-term with a birth weight of 3 kg. The perinatal period was unremarkable.

Physical Examination (at 5 months): Head circumference 43.5 cm, weight 10.5 kg (overweight). Examination revealed horizontal nystagmus, bilateral simian creases, umbilical hernia, generalized hypotonia, diminished tendon reflexes, and bilateral positive Babinski signs. Cardiopulmonary examination was normal.

Follow-up: With symptomatic rehabilitation, he showed developmental progress. He sat momentarily at 11 months, achieved head control at 1 year, sat independently at 2 years, stood independently at 4 years, and walked independently at 4.5 years. He walked with a toe-walking, waddling, unstable gait. By age 7, he could squat and stand but had poor balance and could not run. Language milestones included reduplicated words at 1.5 years and simple 5 to 6 word sentences at 5 years, with dysarthria. Vision and hearing were normal. He understood simple sentences and could communicate but had poor memory and no arithmetic skills. At the last follow-up (age 16 years), he walked independently with a waddling gait but could not run. He maintained basic communication skills.

### 3.2. Neuroimaging and laboratory findings

The proband underwent cranial MRI at 8 months of age, which reportedly showed deepened and widened sulci and fissures (per paternal report; images lost). The brother underwent cranial MRI at 5 months, revealing slightly rounded posterior horns of the lateral ventricles and a thin corpus callosum. Subsequent MRI scans for the brother were unremarkable.

Metabolic screening (blood amino acids, acylcarnitine, urine organic acids) was normal for both siblings. Routine blood work, biochemistry, liver/kidney function, immune profiles, ESR, and myocardial enzymes were normal. Methylation-specific MLPA for Prader-Willi/Angelman syndromes was negative. Karyotype analysis showed a normal chromosomal constitution, and no pathogenic Copy Number Variations (CNVs) were detected.

### 3.3. Genetic findings

Whole-exome sequencing identified identical compound heterozygous variants in HACE1 in both siblings. The paternal variant was c.1303C > T, resulting in a nonsense change, p.Gln435Ter (p.Q435*). This variant introduces a premature stop codon and is predicted to cause loss of the downstream HECT domain. According to ACMG guidelines, it was classified as pathogenic. The maternal variant was c.2158_c.2159insG, resulting in a frameshift change, p.Glu720Glyfs*17 (p.E720Gfs*17). This variant is predicted to disrupt the HECT domain and was classified as likely pathogenic. Sanger sequencing confirmed that the father carried the c.1303C > T variant and the mother carried the c.2158_c.2159insG variant. Both variants were present in the 2 affected siblings and were considered novel, as they had not been previously reported in the reviewed literature or public databases. The sequencing results and protein domain distribution are shown in Figure 1.

**Figure 1.**
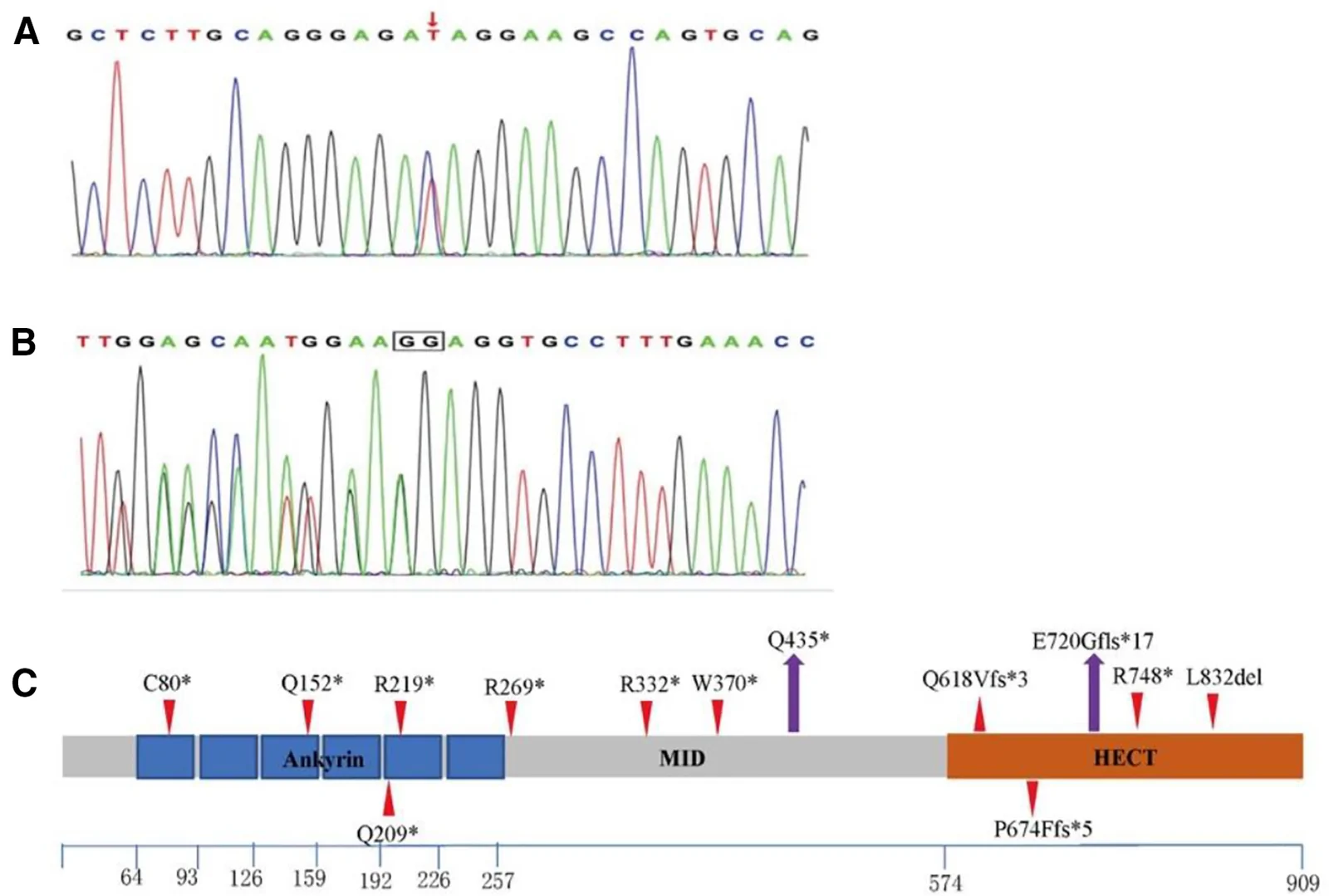
Results of Genetic Analysis of *HACE1*. A: Novel nonsense mutation c.1303C > T (p.Q435*) in exon 12 of *HACE1* (arrow indicates mutation site). B: Frameshift mutation c.2158_c.2159insG in exon 19 of *HACE1* (box indicates inserted G base), p.E720Gfs*17. C: Protein domain structure of *HACE1* (909 aa): Contains 6 ankyrin repeats (blue, 64909aa). MID (middle region, gray). Red triangles indicate previously reported mutations. Purple arrows indicate mutations reported in this study.

### 3.4. Literature review

A systematic search of PubMed, Wanfang, CNKI, and VIP databases using the terms “SPPRS” and “HACE1” from 2015 to 2025 yielded 9 international publications reporting 25 SPPRS patients (see Table S1, Supplemental Digital Content 1).^[1–9]^ The 25 reported patients originated from 14 families; 8 families (57.1%) were consanguineous. The male-to-female ratio was 3:2. Onset occurred between birth and 2 years 8 months, with hypotonia as the universal presenting symptom (25/25). Lower limb spasticity developed in 15 patients (60.0%). Motor development was severely compromised: only 4 patients (16.0%) achieved independent walking (with instability), 4 (16.0%) could move short distances with assistance, and the remainder were nonambulatory. At a median follow-up age of 9.9 years (range: 4 months to 31 years), all patients exhibited psychomotor and significant language retardation. Seizures occurred in 11 patients (44.0%), most commonly myoclonic. Physical features included overweight status in 13 patients (52.0%) and skeletal abnormalities (kyphosis, scoliosis, hip dislocation, foot deformities) in 15 patients (60.0%). Dysmorphic features were noted in 14 patients (56.0%), including macrocephaly (4), microcephaly (4), and facial dysmorphism (8). Ocular abnormalities were present in 12 patients (48.0%), including myopia/strabismus (9) and retinal dystrophy (5). Sensorineural hearing loss was reported in 4 patients (16.0%). Cranial imaging was abnormal in 14/21 patients (66.7%), with cerebral hypoplasia (corpus callosum, white matter, myelination defects) seen in 9 cases and atrophy in 5 cases. Genetically, 21 patients (84.0%) carried homozygous *HACE1* mutations, while 4 (16.0%) were compound heterozygous. A total of 16 mutations were reported; excluding 1 single amino acid deletion, all were truncating mutations. Mutations affected the N-terminal ankyrin repeats in 7 cases, the HECT domain in 6 cases, both domains in 1 case, and the middle region in 4 cases.

## 4. Discussion

Intellectual disability or global developmental delay represents a broad category of neurodevelopmental disorders characterized by high clinical and genetic heterogeneity. Its etiology is complex, involving a multifactorial interplay of genetic and environmental factors. Clinical manifestations are diverse and highly heterogeneous. The prevalence in the global population is approximately 1%, with severe intellectual disability affecting approximately 0.6%.^[10]^ With rapid advancements in genetic technologies, the genetic basis of neurodevelopmental disorders has garnered increasing attention. The phenotypic spectrum is broad and varied, presenting significant diagnostic challenges. SPPRS was first reported in 2015, and understanding of this condition remains limited. Its pathogenic gene is *HACE1*, located on chromosome 6q16.3, which encodes an E3 ubiquitin-protein ligase containing a HECT domain and ankyrin repeats (HACE1).^[11]^ The C-terminal HECT (Homologous to the E6-AP Carboxyl Terminus) domain is a catalytic domain of approximately 40 kDa (574~909 aa), with its active cysteine residue located at position 876. It mediates the interaction between the E3 ligase and substrates through various mechanisms, participating in the subcellular localization and proteasomal degradation of target proteins. The N-terminal ankyrin repeats mediate protein-protein interactions, exerting biological functions by binding to ligands.

Here, we describe 2 Chinese siblings with *HACE1*-associated SPPRS and provide their long-term clinical follow-up. The initial presentation included hypotonia, accompanied by psychomotor retardation and language delay. Cranial imaging data were severely lacking. Upon presentation to our hospital, a definitive diagnosis could not be established. Methylation-specific Multiplex Ligation-dependent Probe Amplification (MLPA) for Prader-Willi Syndrome (PWS)/Angelman Syndrome (AS) and karyotyping excluded PWS, AS, and chromosomal abnormalities. With the progression of molecular genetic testing technologies, particularly the clinical application of Whole Exome Sequencing (WES), an increasing number of novel pathogenic genes are being identified. Compound heterozygous mutations in the HACE1 gene were identified in the 2 children: c..1303 (exon12)C > T and c..2158 (exon19)_c.2159insG. The former was inherited from the father, and the latter from the mother. Neither mutation has been reported domestically or internationally. The rationale for considering these mutations as pathogenic in these 2 patients includes: the parents each carry 1 mutation but are phenotypically normal, transmitting the mutant alleles to their offspring, consistent with an autosomal recessive inheritance pattern; these variants lead to a loss of gene function; conservation analysis and protein structure prediction software (MutationTaster, GERP, phyloP20way, phastCons20way) indicate that the frameshift variant affects protein synthesis and possesses potential pathogenicity; and these nucleotide variants are not classified as polymorphisms in the 1000 Genomes Project database. Consequently, we conclude that the etiology in these 2 patients is compound heterozygous mutations in the *HACE1* gene. When compared with previously reported clinical characteristics of *HACE1*-mutated SPPRS, the 2 patients in this report presented with hypotonia as the initial symptom and global developmental delay. Their clinical phenotype appears relatively milder compared to previous reports; they demonstrated gradual improvement with age, achieving independent ambulation (albeit with a waddling, unsteady gait), acquiring a vocabulary of 5 to 6 words, understanding basic sentences, and engaging in simple communication. The clinical features show a moderate degree of concordance. In summary, combining the *HACE1* gene mutations with the SPPRS clinical profile, the diagnosis of SPPRS in these 2 patients is definitive.

Previous studies have suggested that *HACE1* is a potential tumor suppressor gene, significantly downregulated in various malignancies, including Wilms’ tumor, B-cell lymphoma, and neuroblastoma, and is involved in pathophysiological processes.^[12–15]^ In the Chinese population, *HACE1* gene polymorphisms have also been associated with susceptibility to Wilms’ tumor and neuroblastoma.^[16,17]^ Hibi et al^[18]^ detected aberrant methylation of the *HACE1* gene in 9 out of 32 (28.1%) patients with advanced primary colorectal cancer; these patients exhibited larger maximum tumor areas and a higher risk of lymph node metastasis, suggesting a potential malignant role for *HACE1* methylation in primary colorectal cancer. Similar conclusions have been drawn in studies of primary gastric and hepatocellular carcinomas.^[19,20]^ In vivo animal studies have also demonstrated that *HACE1*^-/-^ mice are more prone to spontaneous, late-onset cancers.^[12]^

Contrary to many studies associating *HACE1* mutations with tumor susceptibility phenotypes, our literature review indicates that neither previously reported SPPRS patients nor carriers have reported malignancies, although the possibility of late-onset tumors cannot be excluded. All patients presented with psychomotor retardation. The 2 patients reported herein were found to harbor 2 novel variants: a nonsense mutation p.Q435* and a frameshift mutation p.E720* (insertion G). As of the last follow-up, clinical features were limited to neurodevelopmental disorders, consistent with previous reports, without evidence of cancer susceptibility. This necessitates further investigation into the loss of *HACE1* function and possible mechanisms underlying neurodevelopment.

Rac1 is a critical member of the Rho subfamily. Its activity is regulated by multiple mechanisms, including ubiquitination and degradation. Studies have found that *HACE1*, acting as an E3 ubiquitin ligase, catalyzes the ubiquitination of Rac1, mediating its degradation. Through migration stimulation, it activates the ubiquitin-proteasome pathway, recognizing and degrading ubiquitinated active Rac1-GTPase. Downregulation of *HACE1* is associated with increased total Rac1 levels and Rac1 accumulation in membrane ruffles.^[21]^ The 64th edition of the Catalogue of Somatic Mutations in Cancer (COSMIC) identified 13 missense mutations outside the HECT domain of *HACE1*, all of which can lead to arrested cell growth and impaired Rac1 ubiquitination.^[22]^ Iimura et al^[23]^ demonstrated in Xenopus laevis that *HACE1* expression is specifically upregulated in the ectoderm during early embryonic blastula and gastrula stages, as well as in the epidermis, branchial arches, kidney, and tail bud stages. Knockout of embryonic *HACE1* resulted in defects in body axis elongation, pigmentation, and eye formation at the tadpole stage, inhibited neural convergent extension, and delayed neural tube closure, suggesting *HACE1* may function by regulating Rac1 activity.^[23]^ Reijnders et al,^[24]^ in 7 cases with de novo missense RAC1 mutations presenting with varying degrees of developmental delay, brain malformations, and other phenotypes, utilized computer simulations of mouse fibroblast spreading assays and in vivo overexpression assays in zebrafish to demonstrate that Rac1 dysregulation can lead to microcephaly, reduced neuronal proliferation, and cerebellar malformations. Research has also indicated that Rac1 participates in cerebellar development by influencing cell morphogenesis, migration, and formation.^[25,26]^ In summary, Rac1 is crucial in early neuronal development; thus, HACE1 plays a potential role in neurogenetic developmental disorders.

Nagy et al^[4]^ first established an SPPRS mouse model by knocking out Hace1, which exhibited intellectual and motor development deficits, ventricular enlargement, corpus callosum agenesis, decreased Rac1 levels, and altered active forms. Concurrently, in vitro experiments using cultured SPPRS patient fibroblasts showed significant upregulation of Rac1-GTPase and promotion of Rac1-regulated downstream pathways, suggesting that *HACE1* knockout leads to reduced synapse numbers and neuropathological features in SPPRS patients. Zhao et al found that *HACE1* inhibits the transcriptional activity of retinoic acid receptor (RAR) isoforms RARα1 and RARβ (isoforms 1, 2, and 3) and may influence neuronal differentiation and regeneration by repressing the activity of RAR-regulated genes such as CRABPII, RIG1, and RARβ2.^[27]^

Studies have reported that *HACE1* is involved in the pathogenesis of hereditary neurodevelopmental diseases such as Alzheimer’s disease and Huntington’s disease.^[28,29]^ In Huntington’s disease, overexpression of *HACE1* promotes the Nrf2 antioxidant response, attenuating oxidative stress induced by mutant Huntingtin protein in vitro.^[30]^ In the Huntington’s disease transgenic *YAC128* mouse model, *HACE1* knockout revealed striatal astrogliosis and significant dysregulation of marker expression, leading to mitochondrial respiratory defects and increased ROS production. This resulted in toxic glutamine accumulation, thereby accelerating motor deficits and exacerbating cognitive and psychiatric impairments.^[31,32]^

Currently, the specific pathogenic mechanisms of *HACE1*-mutated SPPRS remain unclear. They may be related to mechanisms such as *HACE1* targeting Rac1 to regulate downstream pathways, influencing Nrf2 antioxidant and oxidative stress responses, and inhibiting downstream genes of the RAR family. There is no effective curative treatment for SPPRS. Clinically, early detection and diagnosis, followed by early initiation of symptomatic supportive treatment to improve muscle tone and rehabilitation training, are considered primary management strategies. In this study, the proband’s brother was diagnosed earlier and commenced symptomatic rehabilitation training sooner; his clinical improvement and psychomotor progress were significantly superior to those of the proband. The limitations of this study include the inability to obtain cranial imaging data for comparative analysis and the lack of in-depth research on the potential pathogenic mechanisms of SPPRS caused by *HACE1* mutations in patient pathological tissues.

## 5. Conclusion

In conclusion, this study reports 2 patients with *HACE1*-mutated SPPRS. Their phenotypes were similar to previously reported cases but relatively milder, showing improvement with age. This expands the clinical phenotypic spectrum of *HACE1* mutations and provides a basis for genetic counseling and prenatal diagnosis. Currently, the number of reported *HACE1*-mutated SPPRS cases remains small. A deeper understanding of this disease requires further case accumulation. Simultaneously, in-depth research into the mechanisms of *HACE1* in neurodevelopment is necessary to better elucidate the relationship between this genotype and neurodevelopmental disorder phenotypes.

## Acknowledgments

We thank all the family of these children for their participation and cooperation.

## Author contributions

**Conceptualization:** Jiong Deng.

**Writing – original draft:** Jiong Deng.

**Formal analysis:** Yuheng Gao.


