# Supplementary material for: Two siblings presented with spastic paraplegia and psychomotor retardation with or without seizures with HACE1 variants: A case report and literature review
Source: Medicine (Baltimore). 2026 Oct 9;105(41):e51037. doi: 10.1097/MD.0000000000051037 (PMC13657485; doi:10.1097/MD.0000000000051037)
Supplement: Supplementary file 1 [file medi-105-e51037-s001.docx]

Table 1. Clinical Features of 25 SPPRS patients with *HACE1* mutations

| Referenc | | Hollstein et al.^1^ | | | | | | | | Akawi et al.^2^ | | | | | | Hariharan el al.^3^ | Nagy et al.^4^ | | | Reuter, H et al.^5^ | | Ugarteburuet al.^6^ | Valeriia A Kovalskaia et al.^7^ | Gunes Sager et al.^8^ | | Hamd Alzaidan et al.^9^ |
| --- | --- | --- | --- | --- | --- | --- | --- | --- | --- | --- | --- | --- | --- | --- | --- | --- | --- | --- | --- | --- | --- | --- | --- | --- | --- | --- |
| Family ID | | F1 | | | | | F2 | | | F3 (Consanguineous) | | | F4 | F5 (Consanguineous) | F6 (Consanguineous) | F7 (Consanguineous) | F8 (Consanguineous) | F9 (Consanguineous) | | F10 (Consanguineous) | | F11 | F12 | F13 | | F14 (Consanguineous) |
| Case ID | | P1 | P2 | P3 | P4 | P5 | P6 | P7 | P8 | P9 | P10 | P11 | P12 | P13 | P14 | P15 | P16 | P17 | P18 | P19 | P20 | P21 | P22 | P23 | P24 | P25 |
| Gender | | Male | Male | Male | Male | Female | Female | Male | Male | Male | Female | Female | Male | Female | Male | Male | Female | Female | Male | Female | Female | Male | Male | Male | Male | Female |
| Nationality | | Pakistani | | | | | German | | | Arab | | | British | NA | Pakistani | Indian | Saudi Arabian | Turkish | | Syria | | Pakistani | Russia | Turkish | | Saudi Arabian |
| Age of onset | | 3–4 months | | | | | At birth | | | At birth | | | | | | 1 month | At birth | At birth | | NA | NA | Several months after birth | 2y8m | 1-2 month | | 11 months |
| Initial Symptoms | | Hypotonia | | | | | Hypotonia | | | Hypotonia | | | | | | Hypotonia | Hypotonia | Hypotonia | | Hypotonia | | Hypotonia | Hypotonia | Hypotonia | | Hypotonia |
| Last follow-up | Age | 18y | 15y | 22y | 19y | 3y | 10y | 7y | 7y | 3y | 13y | 15y | 31y | 4y | 4y | 11y | 5y | 4m | 6m | NA | NA | 10y | 4y | 8y | 17y | NA |
|  | BMI  /kg.m^-2^ | 33.3 | 37 | 55.7 | 50.4 | 20.6 | 24.8 | 21.2 | 19.3 | NA | NA | NA | Obese | 21.1 | NA | 20.7 | 20.7 | 21.2 | 22.3 | NA | NA | NA | NA | NA | NA | NA |
| Motor function | sitting | NA | | | | | NA | | | 2-2.5y | 5y | 2.5-3y | NA | NA | NA | 6y | 5y | Unable | Unable | NA | NA | Unable | Unable | Unable | Unable | Unable |
|  | Walking | Unable | Unable | 16y：Short distance with walker | 15y：10–15 steps with wheelchair assistance | Unable | 4y：Ataxic gait | 3-4y：Ataxic gait | 4.5y：Ataxic gait | Unable | Unable | Unable | 2.5-3y：Ataxic gait | 4y: Independent standing, unable to walk | 3 y: Independent standing, unable to walk | 11 y: Assisted walking with support | Unable | Unable | Unable | NA | NA | Unable | Unable | 5 y: Assisted walking | Unable | Unable |
|  | Symptoms | Hypotonia, lower limb spasticity | | | | | Hypotonia | | Hypotonia, lower limb spasticity | Hypotonia, mixed lower limb spasticity and dystonia (mild paresis) | | Hypotonia, lower limb spasticity | Hypotonia, lower limb spasticity | Hypotonia, lower limb spasticity | Hypotonia, gradual improvement | Hypotonia, lower limb spasticity | Hypotonia | Hypotonia | | Hypotonia | | Hypotonia，lower limb stiffness | Hypotonia, spastic quadriplegia | Hypotonia, lower limb spasticity | | Hypotonia, lower limb spasticity |
| Cognitive function | | Developmental delay, ID, poor comprehension | | | | | Developmental delay, ID | | | Developmental delay, ID | | | Developmental delay, ID | | | Developmental delay, ID | Developmental delay | Developmental delay | | Developmental delay | | Severe ID | Developmental delay | ID | | Developmental delay |
| Language | | Few words | | | | | Few words | Dysarthria | Normal | 2.5-3y | Unable | 2-2.5y | 3-4y | Few words | 16m | 6y | Few words | Unable | Unable | NA | NA | Only reduplicated words | Unable | NA | NA | Unable |
| Seizures | | Yes | Yes | No | Yes | Yes | No | Yes | No | No | Yes | Yes | Yes | Yes | No | Yes | No | No | No | No | No | No | No | No | Yes | No |
| Other features | Ocular | Divergent strabismus | Divergent strabismus | Right strabismus, myopia, right macular hypoplasia, retinal dystrophy | Divergent strabismus, myopia, bilateral retinal dystrophy | Left strabismus, myopia, retinal dystrophy | Normal | Normal | Convergent strabismus, myopia, retinal dystrophy | Normal | Normal | Nystagmus | Myopia | Normal | Normal | Heterochromia iridis | Retinal dysplasia | Right strabismus | Bilateral strabismus | Normal | Normal | Normal | Convergent strabismus | Normal | Normal | Normal |
|  | Auditory | Bilateral sensorineural hearing loss | Normal | Bilateral sensorineural hearing loss | Normal | Bilateral sensorineural hearing loss | Normal | | | NA | | | Normal | Normal | Normal | Normal | Bilateral sensorineural hearing loss | Normal | | Normal | Normal | Normal | Normal | Normal | Normal | Normal |
| Imaging | Cranial | Cerebral hypoplasia, marked frontal and temporal atrophy | NA | Generalized cerebral atrophy | Normal | Marked generalized cerebral and brainstem atrophy, abnormal gray/white matter ratio | Corpus callosum hypoplasia | Corpus callosum hypoplasia | Ventriculomegaly | Significantly delayed myelination, reduced white matter, corpus callosum hypoplasia, small pons | NA | Significant reduction in white matter, delayed myelination | Normal | Corpus callosum hypoplasia | Corpus callosum hypoplasia | Corpus callosum hypoplasia | Corpus callosum hypoplasia，mild ventriculomegaly | Normal | Normal | NA | NA | Diffuse right temporal atrophy and arachnoid cyst | Severe cerebral cortical atrophy and moderate ventriculomegaly | Normal | Normal | Normal |
|  | Other | / | / | / | Ventricular dilation | / | / | / | / | / | / | / | / | / | / | / | / | / | / | / | / | / | / | / | / | Atrial septal defect |
| Skeletal Development | | Normal | Left talipes equinovarus | Erb's palsy, right subtalar fusion | Calcaneovalgus deformity | Kyphosis, bilateral hip dislocation | Lumbar lordosis | Pes planus | Mild clubfoot | Normal | Kyphosis, bilateral hip dislocation | Scoliosis | Pes planus, small feet, short and thin fingers | Normal | Normal | Lumbar lordosis, proximal tibial dysplasia | Bilateral hip dislocation; thoracolumbar kyphoscoliosis; ulnar deviation of wrists and small feet | Normal | Delayed closure of anterior fontanelle | Normal | | Normal | Transverse palmar creases and joint hypermobility | Normal | Normal | Delayed tooth eruption, microcephaly |
| Facial Dysmorphism | | Macrocephaly | Macrocephaly | Macrocephaly | Macrocephaly | Normal | Normal | | | Normal | Normal | Normal | Downturned mouth, thin lips | Deep-set eyes, long palpebral fissures, microcephaly | Epicanthus, hypertelorism, microcephaly | Normal | Microcephaly and brachycephaly, downturned mouth | Frontal bossing, prominent glabella, short and depressed nasal bridge, high nasal bridge, everted lower lip, high palate, ear dysplasia | Frontal bossing, prominent glabella, short and depressed nasal bridge, high nasal bridge, everted lower lip | Normal | | Normal | Coarse facies, low anterior hairline, thick eyebrows, long eyelashes, long philtrum, short neck, increased eye lines | Microcephaly | | Hemangioma (right forehead and eyelid) |
| HACE1 Mutation | Protein Domain | N-terminal Ankyrin repeats | | | | | HECT domain | | | HECT domain | | | N-terminal Ankyrin repeats, HECT domain | MID | N-terminal Ankyrin repeats | MID | N-terminal Ankyrin repeats | MID | | N-terminal Ankyrin repeats | | N-terminal Ankyrin repeats | N-terminal Ankyrin repeats | N-terminal Ankyrin repeats | | HECT domain |
|  | Mutation Site | p.R219* | | | | | p.R748*（rs869025281）；p.P674Ffs*5 | | | p.L832del | | | p.Q152*，p.Q618Vfs*3 | p.R269* | p.C80* | p.W370* | p.Q209* | p.R332* | | c.402+5G>A | | p.C80* | c.(534+1_535-1)_(617+1_618-1)del | p.Ala861Pro | | p.R664* |
|  | Zygosity | Homozygous | | | | | Compound Heterozygous | | | Homozygous | | | Compound Heterozygous | Homozygous | Homozygous | Homozygous | Homozygous | Homozygous | | Homozygous | | Homozygous | Homozygous | Homozygous | | Homozygous |
| Remarks | | Genital hypoplasia | Genital hypoplasia | Genital hypoplasia | / | Died of pneumonia at age 9 | / | / | / | / | Cardiomyopathy | / | / | / | / | / | Hypothyroidism, adrenal insufficiency | / | / | Recurrent infections | | 3-methylglutaconic aciduria | / | / | / | Dysphagia, recurrent respiratory infections |
